# MUSIC IN THE EVERYDAY LIFE OF PEOPLE LIVING WITH DEMENTIA

**DOI:** 10.1093/geroni/igad104.1400

**Published:** 2023-12-21

**Authors:** Joseph Kotarba, Amanda Couve

**Affiliations:** Texas State University, San Marcos, Texas, United States; Heritage Creek Assisted Living Center, San Antonio, Texas, United States

## Abstract

A common approach to dementia care is the clinical application of music to improve cognition, memory, quality of life, etc. Although the literature on this work continues to expand, relatively little scholarly attention has been given to understanding common, everyday life experiences of music among those living with dementia. The present paper is derived from the qualitative research informing “Music in the Course of Life” (Routledge, 2023). Following the symbolic interactionist/existentialist paradigm, we reconfigured the life course model of organizing aging by seeing life course as a concept of the self that can be observed in various situations, that are not necessarily linearly ordered, in everyday life. We observed music as it is presented and performed in residential facilities, at home, in hospitals and in hospice. The self experience can be portrayed as “Being,” “Becoming,” and “Been There” in the case of many seniors. The primary value of music for dementia care is not predicated on eliciting music from memory or from regenerating cognition. The pleasures of music are exceptionally situational so that the elderly person need not have any prior exposure to or experience with music that now is positive affectively, cognitively and/or socially. The meaningful music situation--especially at end of life--is composed of significant others providing the music, attendees singing or humming along with the music, a caretaker to orchestrate the situation.

